# Study design and rationale for a cluster randomized trial of a safe child feces management intervention in rural Odisha, India

**DOI:** 10.1186/s12889-021-12405-0

**Published:** 2022-01-15

**Authors:** Gloria D. Sclar, Valerie Bauza, Hans-Joachim Mosler, Alokananda Bisoyi, Howard H. Chang, Thomas F. Clasen

**Affiliations:** 1 Gangarosa Department of Environmental Health, Rollins School of Public Health, Emory University, Atlanta, GA USA; 2 Department of Psychology, University of Zürich, Zürich, Switzerland; 3 RanasMosler, Zürich, Switzerland; 4 Independent Consultant, Berhampur, Odisha India; 5 Department of Biostatistics and Bioinformatics, Rollins School of Public Health, Emory University, Atlanta, GA USA

**Keywords:** Child feces, Safe disposal, Latrine training, Behavior change, Sanitation, Theory-based intervention

## Abstract

**Background:**

Poor child feces management (CFM) is believed to be an important source of exposure to enteric pathogens that contribute to a large disease burden in low-income settings. While access to sanitation facilities is improving, national surveys indicate that even households with latrines often do not safely dispose of their child’s feces. Working with caregivers in rural Odisha, India, we co-developed an intervention aimed at improving safe disposal of child feces and encouraging child latrine use at an earlier age. We describe the rationale for the intervention and summarize the protocol for a cluster randomized trial (CRT) to evaluate its effectiveness at changing CFM practices.

**Methods:**

The intervention consists of six behavior change strategies together with hardware provision: wash basin and bucket with lid to aid safe management of soiled nappies and a novel latrine training mat to aid safe disposal and latrine training. The intervention will be offered at the village level to interested caregivers of children < 5 years of age by a community-based organization. Following a baseline survey, 74 villages were randomly allocated to either intervention or control arm. The primary outcome is caregiver reported safe disposal of child feces after last defecation, either by the caregiver disposing of the child’s feces into the latrine or the child using the latrine, measured approximately four to six months following intervention delivery. Secondary outcomes include fecal contamination of household drinking water and the childs’ hands. A process evaluation will also be conducted to assess intervention fidelity and reach, and explore implementer and participant feedback.

**Discussion:**

This study addresses a crucial knowledge gap in sanitation by developing a scalable intervention to improve safe management of child feces. The behavior change strategies were designed following the Risks, Attitudes, Norms, Abilities and Self-Regulation (RANAS) approach, which has shown to be effective for other environmental behavior change interventions in low-income settings. The latrine training mat hardware is a novel design developed cooperatively and manufactured locally. The evaluation follows a rigorous CRT study design assessing the impact of the intervention on CFM behavior change, as well as fecal contamination of two sources of potential exposure.

**Trial registration:**

This trial is registered at ISRCTN: ISRCTN15831099.

**Supplementary Information:**

The online version contains supplementary material available at 10.1186/s12889-021-12405-0.

## Background

Safe sanitation is critical for ensuring individuals and communities can lead healthy lives [1]. As such, Sustainable Development Goal (SDG) 6.2 seeks to *“achieve access to adequate and equitable sanitation and hygiene for all and end open defecation”* by 2030 [2]. Over the past decade the world has made great progress in increasing access to at least basic sanitation and reducing open defecation, with only 9% of the global population open defecating as of 2017 [3]. However, an often overlooked component of sanitation is safe child feces management (CFM). In an analysis of Demographic Health Survey data (2005–2014) from 34 low- and middle-income countries, an estimated 50.6% of households with a child < 5 years old did not hygienically dispose of their child’s feces into a latrine or have the child use the latrine [4].

While increased latrine coverage has reduced contamination of the environment from adult open defecation, poor child feces management (CFM) may pose a risk to health. Firstly, children’s feces may contain more pathogens compared to adult feces [5]. Due in part to immature immune systems, young children have the highest incidence of enteric infections which may lead to higher pathogenic loads in their feces [6]. Furthermore, household members and neighbors are at greater risk of exposure to child feces because young children typically defecate around the household compound and their feces are disposed of near the household [7]. Research has also found that young children’s feces are a more common source of fecal contamination in households than the feces of older children or adults [8]. This exposure risk is even greater for other young children who spend large amounts of their time on the ground and often engage in exploratory behaviors, including putting fingers, fomites, and soil in their mouths [9, 10]. Lastly, how caregivers manage their child’s feces consists of a string of practices that result in multiple points of exposure, such as the area where the child defecated, the material used to pick up the feces, where the feces are disposed, and whether or not the caregiver washes her and the child’s hands afterwards [11, 12]. Consistent with this evidence of increased exposure from poor CFM, studies have shown that *improved* child feces disposal practices are associated with reductions in diarrhea as well as child stunting and underweight [4, 7].

India in particular has low rates of safe disposal of child feces, despite the country’s significant gains in latrine coverage from decades of national sanitation campaigns. The latest National Family Health Survey (2015–2016) reported only 36% of Indian households safely disposed of their child’s feces into a latrine the last time the child defecated, despite 61% of households having access to a latrine [13]. The State of Odisha had the lowest rate at 13% safe disposal of child feces with 35% latrine coverage [13]. A cross-sectional survey examined child feces disposal practices in rural Odisha following the government of India’s Total Sanitation Campaign and found that while 78.6% of the children resided in a home with a latrine, only 22.8% had their feces safely disposed of into the latrine [14]. Another study in Odisha reported that while a community-based water and sanitation program increased adult latrine use to 74%, only 35% of children < 5 years old had their feces safely disposed [15].

There is a need for effective behavioral interventions that focus on safe CFM practices among caregivers, with the eventual goal of the child learning to use the latrine. Some maternal and child health interventions and water, sanitation and hygiene (WASH) interventions include aspects of child feces disposal, but it is typically one of several target behaviors and is addressed through health education messaging with nominal grounding in behavior change theory [16, 17]. Moreover, evaluations of these interventions sometimes only measure health outcomes, making it unclear how effective they are at actually changing CFM behaviors [16]. Two exceptions are the recent efficacy trials of the SHINE (Sanitation Hygiene Infant Nutrition Efficacy) and WASH-Benefits studies. The SHINE CFM intervention in Zimbabwe specifically targeted safe disposal of wash water from cleaning infant nappies for children < 18 months, reporting 77% safe disposal among the WASH trial arms compared to 32% among the non-WASH arms [18]. The WASH-Benefits trial in Kenya provided a sani-scoop tool for feces removal and a plastic potty for children < 3 years old, and reported 33–37% safe disposal in the sanitation and WASH arms compared to 10% in the control arm at the two-year follow-up [19]. The WASH-Benefits trial in Bangladesh reported moderate use of the potties and low use of the sani-scoops based on structured observations [20]. Both the SHINE and WASH-Benefits trials, however, reported no effect of the WASH intervention arms on child stunting and mixed effects on diarrhea [18, 19, 21]. These results highlight a crucial need for more comprehensive and effective CFM interventions that promote a variety of safe disposal practices for children at different developmental stages (i.e. infant, baby, toddler), *and* that address the related behavioral process of latrine training for eventual child latrine use.

We seek to fill this knowledge gap by demonstrating an approach for developing and evaluating an intervention aimed at improving safe disposal of child feces and child latrine use. The intervention is geared towards primary caregivers of children < 5 years old, and also engages other household members through social support techniques. The intervention was designed based on formative research and following the Risks, Attitudes, Norms, Abilities, and Self-Regulation (RANAS) approach to systematic behavior change [22]. We also employed the user-centered design (UCD) methodology to develop hardware that can aid caregivers in their practice of safe disposal, as well as training young children how to use the latrine for defecation at an earlier age. Gram Vikas, a local community-based organization, is the implementing partner and we will engage villages that previously participated in its water and sanitation program so all caregivers have an enabling WASH environment to perform the target behaviors.

Here we describe the design of a cluster randomized trial (CRT) to evaluate the effectiveness of the intervention at improving two behaviors of interest: safe disposal of child feces and child latrine use. Our primary research question asks: does participation in the intervention increase the practice of safe child feces disposal and child latrine use among households with children < 5 years in rural villages of Odisha with latrine and piped water coverage? We also seek to address the following secondary research questions: (i) Which behavioral factors does the intervention impact and how much does each factor contribute to an increase in safe disposal of child feces and child latrine use? (ii) Is the intervention effective at bolstering social support for primary caregivers when it comes to CFM? (iii) Do the CFM practices promoted in the intervention reduce household fecal contamination compared to current CFM practices? (iv) What aspects of the intervention worked well and what did not work well, from both an implementer and participant perspective? This protocol follows the SPIRIT guidelines [23] (see Supplemental File 1) and the trial is registered at ISRCTN: ISRCTN15831099.

## Methods

### Study design

Evaluation of the CFM intervention follows a cluster randomized controlled trial design. Following a baseline survey, we used stratified randomization to allocate 74 villages to either the intervention or control arm on a 1:1 basis. Approximately four to six months after intervention delivery, we will conduct an endline survey in the study villages among all eligible consenting caregivers of children < 5 years old (at the time of intervention delivery), along with environmental sampling in a sub-set of households to measure fecal contamination. Effectiveness of the intervention will be assessed on an intention-to-treat basis by comparing the proportion of households in intervention and control villages that report safely disposing of their child’s feces (either by the caregiver disposing of the child’s feces into the latrine or the child using the latrine) the last time the child defecated. A process evaluation will be conducted during intervention delivery to provide targeted feedback to the implementing team and to measure intervention fidelity and reach. We will also conduct in-depth interviews post implementation with the Gram Vikas mobilizers and a sub-set of caregivers who participated in the intervention to elicit feedback on what they liked and did not like about the intervention and to identify recommendations for future implementations.

### Study setting and population

The study will take place in Ganjam and Gajapati districts, an area in which the implementing partner Gram Vikas works and where a previous matched-cohort evaluation found low reports of safe disposal [13]. Both districts are predominantly rural (78% in Ganjam, 88% in Gajapati) with agriculture as the main occupation [24]. However, the districts differ in both their geography and demography. Ganjam district includes a varied geography of hills, valleys, coastal plains, and tableland where the vast majority of the population is Hindu (98.8%), only 3.4% belong to the Scheduled Tribe caste category, and 58.7% of rural women are literate [13, 24]. In contrast, Gajapati district is mostly hilly with a population that is 61% Hindu and 38% Christian, the majority (54.3%) are Scheduled Tribe, and 42.1% of rural women are literate [13, 24].

We specifically engaged rural villages that previously participated in Gram Vikas’s community-based water and sanitation program known as MANTRA (Movement and Action Network for Transformation of Rural Areas) [25]. In the MANTRA program the community is mobilized to construct latrines for each household (twin-pit pour flush latrines with attached bathing room) and once this is accomplished then Gram Vikas constructs a community water tank that provides piped water to all households. A sub-study from the matched cohort evaluation examined the CFM practices of caregivers of children < 5 years residing in MANTRA villages and who had access to improved sanitation [26]. The study found that 40.7% of caregivers reported safely disposing of their child’s feces into the latrine the last time the child defecated, with the majority of this safe disposal (84.7%) being a result of the child directly using the latrine. This is in contrast to the overall State of Odisha where only 13% of households practice safe disposal [13].

### Inclusion/exclusion criteria

Villages were eligible for inclusion if they met the following criteria: completed MANTRA program, village size is between 35 to 250 households, 75% of households have access to a latrine, community water tank is functional, village has its own *Anganwadi* center (government-run daycare and preschool center), and Gram Vikas has no programming planned in the village during the study period. The WASH related criteria are meant to ensure most eligible households in the trial villages already have access to water and a latrine so that caregivers have an enabling environment to perform the safe CFM practices being promoted. Moreover, Gram Vikas plans to continue addressing WASH infrastructure needs in villages with lower coverage and functionality, and as such desired the CFM intervention to be tailored towards villages that did not require this additional infrastructure investment. The remaining criteria aim to mitigate issues related to logistics (smaller villages may have no or very few eligible households with young children, larger villages require more inputs), spillover (an *Anganwadi* center used by multiple villages could lead to spread of intervention messaging), and exogenous influences (other Gram Vikas programs could influence effectiveness of the CFM intervention). In addition, eligible villages had to be accessible throughout the year and not previously engaged in formative research activities or piloting of data collection tools.

For the baseline survey, all households with a latrine and at least one child < 5 years old residing in the home were eligible for inclusion. For the endline survey, all households with a latrine and at least one child who was < 5 years old at the time of intervention delivery will be eligible for inclusion. This ensures all households that could have been exposed to the intervention will be attempted for the endline survey. We will only engage participants age 18 years or older for all research activities, except for hand-rinse samples which will engage children < 5 years old upon caregiver consent.

### Intervention

#### Intervention design

The CFM intervention was developed following the RANAS approach to systematic behavior change [27]. Several studies have examined the effectiveness of RANAS for designing behavior change interventions and shown positive impacts on various WASH behaviors including handwashing, safe water consumption, solar water disinfection, and cleaning of shared sanitation [28–34].

**Fig. 1:**
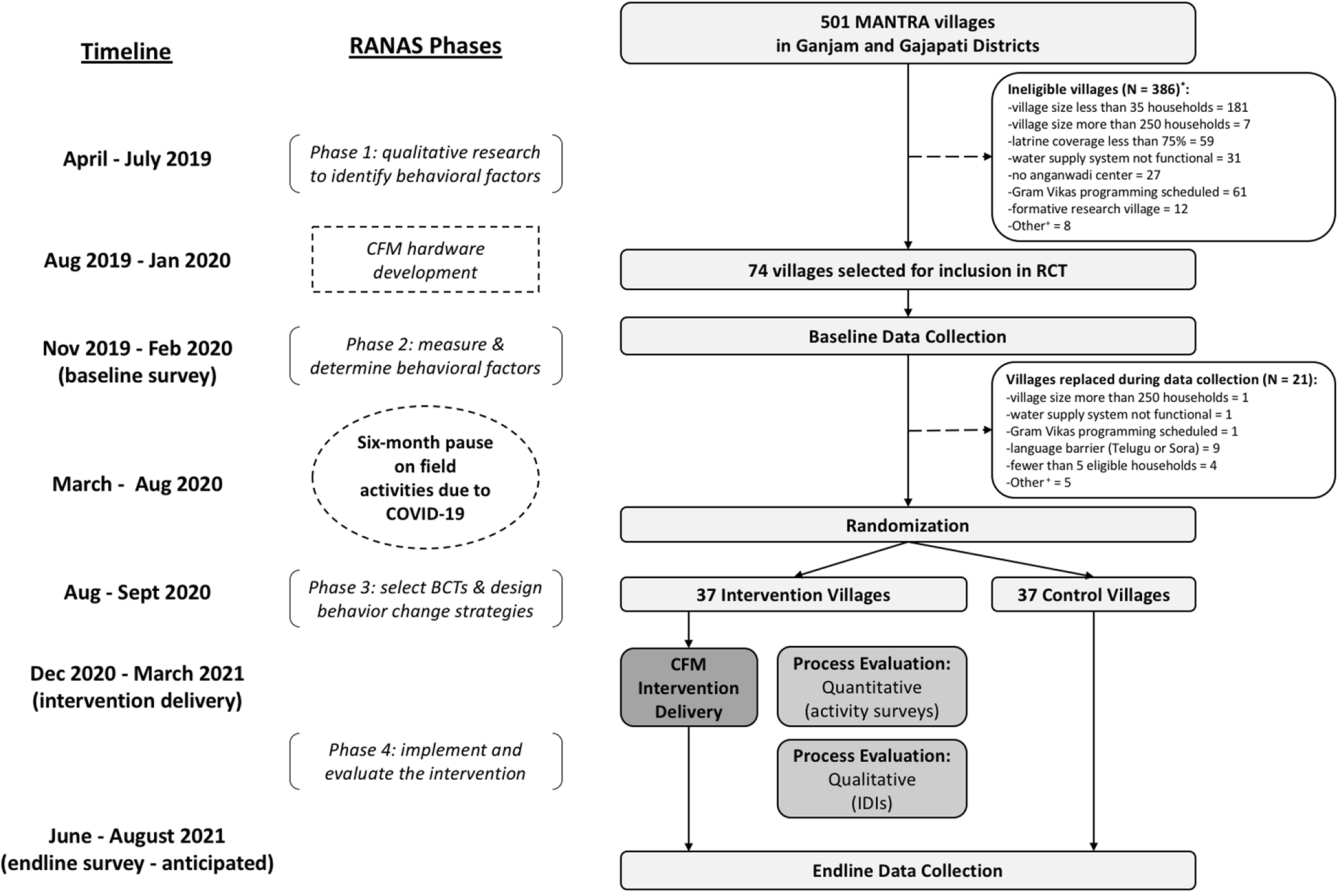
Flow diagram for a cluster randomized trial of a safe child feces management intervention in rural Odisha, India. ^+^Ineligible villages: ‘Other’ (first round) = 5 language barrier (Telugu), 1 fewer than five eligible households, 2 indicated did not want to participate; ‘Other’ (second round) = 4 no longer existed (turned into marketplace, destroyed by cyclone Titli), 1 indicated did not want to participate

The RANAS approach acted as both the underlying *behavioral theory* guiding intervention design as well as the *method* for intervention design. The RANAS behavioral theory outlines five psychosocial factors that may influence a given health behavior — risks, attitudes, norms, abilities, and self-regulation — and also considers contextual factors, such as the physical environment. We used the RANAS theory to examine and address two behaviors: safe child feces disposal and child latrine training. We chose to focus on latrine training, rather than child latrine use itself, because training is a necessary precursor for a child before they can consistently and independently use the latrine. The RANAS approach includes four phases to intervention design with the final phase being an evaluation of the developed program. Here we describe how we designed the CFM intervention in collaboration with Gram Vikas, following phases 1 to 3 (see Fig. 1 for a timeline of the RANAS phases):

- Phase 1 – explore behavioral factors
  Conducted qualitative formative research with Gram Vikas staff to identify potential behavioral factors influencing safe disposal and child latrine training. Research activities included in-depth interviews and focus group discussions with mothers, fathers and grandmothers of children < 5 years across eight villages in the study area.
- Phase 2 – identify target behavioral factors:
  Applied the qualitative findings to construct a structured RANAS questionnaire to measure behavioral factors related to safe disposal and child latrine training. Administered the questionnaire during baseline data collection. Analyzed the data using a ‘doer vs. non-doer’ approach and identified a set of factors steering each behavior of interest.
- Phase 3 – design behavior change strategies:
  Selected behavior change techniques (BCTs) that address the identified factors and drafted potential behavior change strategies. Held a design workshop with Gram Vikas to revise the draft strategies based on implementer feedback and insight. Gram Vikas subsequently pilot tested a subset of the BC strategies in three villages over a two-week period to further revise and finalize the intervention.

Due to the COVID-19 pandemic [35], study activities halted for a six-month period between phases 2 and 3 of intervention design. When study activities resumed with the design workshop, it was decided that the intervention would not include any community-level activities such as a community-wide meeting or edutainment performance, to reduce risk of viral transmission among study participants. In addition, the Gram Vikas team would follow recommended preventative measures such as mask-wearing and keeping physical distance from participants when implementing activities.

#### Hardware development

Behavior change programs sometimes include the provision of hardware in order to assist people in their ability to adopt the promoted behavior. When it comes to the RANAS approach, hardware provision is a specific behavior change technique that falls under the ‘ability’ factor. We decided from the start that the intervention should include provision of CFM hardware to help aid caregivers in their practice of safe disposal and latrine training. The hardware development process took place over a 6-month period prior to phase 3 of the RANAS approach so that the hardware could be incorporated into the behavior change strategies developed during the design workshop.

The hardware was developed through an iterative process that included multiple rounds of user-centered design (UCD) sessions with primary caregivers and other stakeholders from formative research villages. The aim of the UCD sessions was to: 1) elicit participant feedback on locally available CFM hardware, such as plastic potties and scoops, including ways the hardware could be improved upon and 2) co-design novel ideas for CFM hardware with participants. The sessions were followed by distribution and pilot testing of different hardware variations among purposively selected households with young children. Pilot testing was done in two rounds so that hardware could be redesigned and retested based on participant feedback from the first round. Details of the specific hardware development and selection process will be published in forthcoming articles.

Considering pilot findings and age-specific CFM needs, the final selected hardware included locally available wash basins and buckets with lids and a novel latrine training mat with tray. The wash basins and buckets with lids are meant for caregivers who have their child defecate on cloth or use cloth nappies (typically babies between 0 to < 7 months old). The caregiver can use the bucket with lid to safely store soiled cloths and use the basin to safely wash them and dispose of the dirty wash water into the latrine.

The latrine training mat with tray is meant for a wider age group (between 7 to < 48 months old) and can be used as a child grows and eventually learns to use the latrine. For younger toddlers who are not yet ready to start latrine training, the mat can be placed on the ground with a tray underneath it for the child to squat over and defecate (like a squatting version of a sitting potty). When the child and caregiver are ready to start latrine training, the tray can be removed and the mat can be placed over the latrine to create a smaller squat hole that is more child-friendly. Overtime, the caregiver can then transition the child to using the latrine without the mat.

#### CFM intervention - behavior change strategies

The resultant CFM intervention includes six BC strategies (i.e. program activities), which consist of small group meetings and household visits that employ specific BCTs. The target participant for the strategies are primary caregivers, typically mothers, of children < 5 years. However, in some of the strategies other household members, such as fathers and grandmothers, are also engaged to foster a supportive household environment towards the new CFM behaviors. A team of Gram Vikas mobilizers will implement the activities across the 37 trial intervention villages. Due to the nature of the intervention, participant blinding will not be possible. There is no plan to implement the intervention in control villages at the end of the trial. However, Gram Vikas may choose to do so, or implement a modified version of the intervention based on study findings.

The intervention starts with an opening meeting that uses risk, attitude, and norm BCTs and also includes distribution of CFM hardware as an ability BCT. The intervention then shifts to alternating household visits and caregiver group meetings, which primarily use ability and self-regulation BCTs. The final strategy is a closing celebratory meeting that uses norm BCTs. Throughout the intervention, caregivers receive behavioral messaging on how to safely manage their child’s feces based on their child’s current developmental stage. Caregivers will also receive messaging on what to do as their child grows, such as transitioning from safe disposal to latrine training. Each BC strategy is further described below:

- **Hardware and Action Knowledge Opening Meeting:** The meeting starts with a discussion on typical CFM practices and why they are unsafe, followed by a video that tells the story of two mothers; one mother safely manages her child’s feces and another does not, illustrating messages related to health risks, costs and benefits, and the needs at different child development stages. The Gram Vikas mobilizer then uses a banner with illustrations to explain how to use the CFM hardware to safely dispose of children’s feces or teach them how to use the latrine. Volunteers are called upon to demonstrate the new information and then certain hardware is distributed to each caregiver depending on her child’s age (wash basin and bucket with lid for 0 to < 7 months old; latrine training mat with tray for 7 to < 48 months old). The meeting closes with a group commitment to use the new hardware and practice safe disposal and/or child latrine training.
- **Building Self-Efficacy and Goal Setting Household Visits:** The Gram Vikas mobilizer then visits each caregiver at her home and consults with them on their new practice, tailored to safe disposal and/or latrine training. During the visit the caregiver demonstrates her current practice, discusses any challenges she is facing and creates a barrier plan or is given tips, and creates a ‘goal tracker’ to monitor her progress in reaching the behavioral goal. The visit ends with the Gram Vikas mobilizer inviting other household members to express their approval of safe disposal/latrine training and to explain how they will support the caregiver. The second household visit is similar to the first but involves checking on the ‘goal tracker’ and having the caregiver positively self-reflect on her change.
- **Caregiver Support Group Meeting:** Facilitated group meeting is held in-between the household visits to allow caregivers to reflect on their progress, re-commit to their goal of practicing safe disposal/latrine training, and provide strategies to fellow caregivers on how to address common challenges and offer words of encouragement to each other.
- **Celebrating ‘Safe CFM Families’ Closing Meeting:** The final activity is a celebratory meeting that invites caregivers, their family members, and important village stakeholders (e.g. Anganwadi worker) to come together and share their experiences with adopting the safe CFM practices and its importance. The village stakeholders then give each caregiver a certificate to acknowledge her and her household’s achievement.

### Study outcomes and measures

The primary outcome is a binary measure of *safe disposal of child feces,* as defined by the WHO/UNICEF Joint Monitoring Programme for Water Supply and Sanitation (JMP). The updated JMP definition describes safe disposal as encompassing two distinct behaviors based on the last time the child defecated: 1) child used the toilet/latrine or 2) caregiver put/rinsed the child’s feces into the toilet/latrine [36]. JMP defined safe disposal of child feces is assessed in both the baseline and endline cross-sectional samples. The primary caregiver of the child < 5 years old (< 5 years at time of survey for baseline; < 5 years at time of intervention delivery for endline) in the household is asked “The last time [NAME OF CHILD] defecated, where did [NAME OF CHILD] defecate?” If the caregiver reports anything except “in latrine” then the following question is asked “Where was [NAME OF CHILD]‘s feces disposed?” A child’s feces are deemed safely disposed if the child defecated in the latrine or if the caregiver disposed of the child’s feces into the latrine.

We recognize that the primary outcome is subject to response bias since it is measured using caregiver self-report. However, asking a respondent to report on the “last time” a behavior took place rather than the “usual” practice is a more robust approach, as demonstrated for other sanitation practices such as latrine use [37]. This is also the approach taken by the JMP. If a caregiver reports “do not know” for the two questions assessing safe disposal, however, then the caregiver will be asked to report on the “usual” practice instead. We anticipate this case to be rare based on previous research conducted in Odisha [26].

Secondary outcomes include household fecal contamination measured by *E. coli* enumeration from collected drinking water and hand rinse samples; other CFM practices such as material used to handle child feces; child latrine use among children up to age 10; psychosocial factors related to safe disposal and child latrine training measured by RANAS questionnaires; and caregiver received social support for CFM. Both the RANAS and social support questionnaires were developed based on qualitative formative research and revised through cognitive interviews. The social support items measure informational, instrumental, and emotional support pertaining to CFM and were adapted from several validated metrics [38–42]. These secondary outcomes are also assessed at both baseline and endline.

### Sample size

#### Trial

The sample size calculation used to determine the number of trial villages was based on the primary outcome of interest: caregiver-reported safe disposal of child feces during the child’s last defecation event for children < 5 years. We used a version of formula five from Rutterford, Copas [43] for a standard parallel-group, two-arm study design with a binary outcome and cluster-level analysis. Formula parameters came from Gram Vikas provided data and previous sanitation studies conducted in Odisha. We estimated an average of 9 eligible households per village (i.e. households with a latrine and at least one child < 5 years) using Gram Vikas village-level survey data and initial baseline data. The baseline prevalence of safe child feces disposal was estimated to be 40.7% based on the matched cohort sub-study in 2015 to 2016 which examined CFM practices of caregivers residing in MANTRA villages in Ganjam and Gajapati districts, the same context as this trial [26]. An intra-cluster correlation (ICC) of 0.103 and 10% loss to follow-up were derived from a sanitation behavior change trial conducted in Puri district, Odisha in 2017 to 2019 [44]. Lastly, we selected a 15% increase in safe child feces disposal as both a meaningful and reasonable level of behavior change. With a baseline prevalence estimate of 40.7%, a 15% change indicates a behavioral shift where the majority of caregivers practice the desired behavior. Assuming an average of 9 eligible households per village, 40.7% baseline prevalence of safe child feces disposal, an ICC of 0.103, 10% loss to follow-up, 80% power, and a significance level of 0.05, we calculated a sample size of 37 villages per arm to detect a minimum 15% change in the primary outcome (corresponding to a prevalence ratio of 1.37). However, we realized during baseline data collection that the cluster sizes greatly varied. We accounted for this variation in cluster size by repeatedly resampling 37 villages per arm from the selected 74 villages. The median effect size that can be detected with 80% power is 16% across simulations. The sanitation behavior change trial in Puri achieved a 15.2% change in safe child feces disposal, making a 16% change attainable [45].

#### Environmental sampling

For environmental sampling, the sample size will be based on logistical feasibility to ensure that samples can be processed in the laboratory on the same day of collection. As a result, only villages that have a driving time from the laboratory of two to three hours or less will be included in sample collection. Additionally, sample collection will be limited to a maximum of 15 households per village. In villages that have more than 15 households with children < 5 years, households for sample collection will be randomly selected. The procedure followed at endline will be similar to the procedure followed at baseline.

#### Process evaluation

Surveys will be completed in a subset of intervention villages, dependent upon resources and travel logistics, to document fidelity and reach for each type of intervention activity delivered. In-depth interviews (IDIs) will be conducted with both Gram Vikas mobilizers and participants to capture their perspective and feedback on the intervention, as well as contextual factors that impacted delivery. Approximately 30 caregivers and each Gram Vikas mobilizer will be interviewed. More or fewer participants may be interviewed depending on saturation of findings. The caregivers will be purposively selected based on certain characteristics, such as their child’s age, in order to capture a range of perspectives. To act as a check on delivery and as another measure of reach, the endline trial survey will include questions for intervention households about participation in the activities. To assess spillover, control households will be asked questions about whether or not any activities related to CFM took place in their village and if they have ever heard of or seen a latrine training mat.

## Village selection and random allocation

We randomly selected 74 villages from a sample frame of 501 MANTRA villages in Ganjam and Gajapati districts. Following baseline data collection, author GDS used stratified randomization to allocate villages into intervention or control arm on a 1:1 basis (Fig. 1). A computer-based random number generator was used to complete the allocation. Villages were categorized into one of five distinct geographic-demographic (‘geo-demo’) groups, which served as the stratification variable to ensure balance on a wide range of factors. Villages in each ‘geo-demo’ group have a distinct set of characteristics with regard to their geography (coastal, hilly, tableland, etc.), predominant religion and caste, village size, market access and other characteristics. The ‘geo-demo’ groups mostly align with the ‘block’ level administrative unit (a district subdivision), with several blocks falling under each group. As such, three of the ‘geo-demo’ groups are located entirely in Ganjam district while the remaining two are in Gajapati district. We used replacement re-randomization to ensure balance on two a priori specified variables: number of eligible households per village and village-level baseline reported safe disposal of child feces. We used the Mann-Whitney U Test to test if the distribution of villages on these two variables was not significantly different between the study arms. We also used an independent samples t-test to test if the mean baseline reported safe disposal of child feces was not significantly different between the study arms. The a priori balance criteria were met with the first randomization attempt. Participants and study investigators will not be blinded to village treatment assignment.

### Data and sample collection

#### Trial

Baseline and endline data collection will be carried out by a team of enumerators and supervised by a local research manager. All team members will be fluent speakers of the local language Odia. For baseline data collection, we conducted a week-long training followed by several days of pilot-testing in non-trial villages, and the same will be done before endline. Throughout the training, team members provide critical feedback on both data collection logistics and the survey tool, especially with regard to question construction (ease of comprehension, colloquial phrasing, etc.), Odia translation, skip patterns, and more (see Supplemental File 2 for the baseline survey tool). For endline data collection, the team will not be informed of village intervention status, but it may be possible for them to infer due to the visible components of the intervention or survey questions related to the intervention.

During both baseline and endline data collection, the enumerator teams will attempt to survey all eligible households within the study village. The target respondent is the primary caregiver of the child < 5 years old. If the primary caregiver is not available then a secondary caregiver is asked to participate. After confirming eligibility and obtaining verbal consent from the participant, the enumerator will conduct the survey. Following the survey, the enumerator will ask to observe the household’s latrine and bathing room and complete a structured spot-check.

#### Environmental sampling and lab analysis

Household drinking water and hand rinse sample collection will be carried out during the baseline and endline data collection period by a team of enumerators trained in sterile collection techniques. For drinking water samples, enumerators will instruct caregivers to retrieve drinking water in the way that they would for a young child and pour it into a sterile Whirl-pak bag (Nasco, Fort Atkinson, WI, USA). Drinking water storage containers (if applicable) will also be observed by enumerators and any water treatment practices for the sampled water will be recorded. For hand rinse samples, a sample will be collected from the youngest child in the household who is < 5 years old, upon caregiver consent. If the youngest child is not available, then the hand rinse sample will be taken from an older child in the household who is also < 5 years old. If no child is available or the caregiver does not consent, the hand rinse sample will be requested from the caregiver. Prior to sample collection, the enumerators will observe the presence of any dirt on hands or under fingernails and record how much time has passed since the hands were last washed. To collect the hand rinse sample, the person being sampled will be asked to put each hand, one at a time, in a Whirl-pak bag pre-filled with 200 ml of sterile distilled water for 20 s while the enumerator massages the hand through the plastic bag. Collected samples will be stored in a cooler with ice packs for transport back to the laboratory. The samples will then be processed by trained lab technicians using membrane filtration techniques, and plates will be incubated to enumerate *E. coli* in the collected samples.

#### Process evaluation

A separate team of Gram Vikas staff will be trained by the Emory research team to document intervention activities. The staff member will sit-in on the activity and discretely fill-out a survey based on their observations, recording participant engagement and whether each specific activity step was completed. As part of a data quality check, a trained Emory research assistant (RA) will also aim to observe at least one activity observed by each Gram Vikas data collector for each of the three meeting intervention activities.

The Gram Vikas mobilizers will also be trained by the Emory research team on two documentation tools: a record book and household visit behavior surveys. Mobilizers will track participant attendance at each activity they deliver, as well as other activity details, in a record book and will also fill out a brief behavior survey with caregivers at each household visit to track aspects of hardware use and behavioral adoption.

Following completion of the intervention, IDIs will be conducted with Gram Vikas mobilizers and caregivers who participated in the activities. A trained Emory RA will interview each Gram Vikas mobilizer to understand their experience with implementing the activities, what went well, what did not go well, and recommendations for the future. In a similar fashion, a team of trained RAs will conduct the IDIs with caregivers to capture their experience and feedback on the intervention activities.

### Data management

The baseline, endline and process evaluation surveys will be programmed for mobile data collection using the open source software Open Data Kit (ODK) [46]. Programmed skip patterns, numeric constraints for integer answers, and required response (with a ‘refusal’ response option) will ensure data quality and prevent missing data. The surveys will be administered using the ODK Collect application on an encrypted and password-protected Android phone. Completed surveys will be uploaded to a password-protected server that compiles the data. When ready for analysis, data will be downloaded from the server and saved to a password-protected cloud-based folder. Some research activities, such as the environmental sampling, will involve data collection using paper and pen. The data will be entered into an Excel database and the paper surveys scanned to create a digital copy, with all files saved in the secure cloud-based study folders.

IDIs will be audio-recorded upon participant consent. Recordings will be transcribed directly into English and the transcripts de-identified to ensure confidentiality. The audio-recordings and transcripts will be saved in the secure cloud-based study folders.

Only members of the research team will have access to the secure cloud-based study folders.

### Data analysis

#### Trial

We will employ an intention-to-treat analysis for all primary comparisons between the intervention and control arms. The effect of the intervention on JMP defined safe disposal will be assessed using a log-binomial model, which will yield the prevalence ratio of post-intervention safe disposal among intervention households relative to control households. We will use generalized estimating equations (GEE) with robust standard errors to account for village-level clustering and will adjust for stratified randomization by geo-demo group and baseline prevalence of safe disposal. If any relevant variables are found to be substantially imbalanced between the two arms at baseline then an additional model will adjust for these variables. All adjusted and unadjusted models will be reported. GEE will also be used to examine the impact of the intervention on secondary outcomes using the appropriate distribution and link function for each specific secondary outcome. If there is variation in the level of household participation in intervention activities, then a per-protocol analysis will be conducted to better measure the efficacy of the intervention on safe disposal behavior change under ideal conditions. A sensitivity analysis will also be conducted to examine differences in safe disposal between those households that were surveyed at baseline and endline compared to those only surveyed at endline. Lastly, we will employ mediation analysis [47] to examine which behavioral factors the CFM intervention influenced and which behavioral factors were then linked to changes in safe CFM behaviors, including safe disposal and child latrine training.

#### Environmental sampling

We will also use GEE models with robust standard errors that account for village level clustering to analyze if the intervention had an impact on environmental contamination based on collected environmental sampling data from drinking water and hand rinse samples. Separate models will be created for drinking water contamination and hand contamination. Separate models will also be run to analyze the binary outcome of prevalence of *E. coli* as well as the continuous outcome of log *E. coli* concentration. *E. coli* concentration data will be log transformed prior to analysis. If any baseline imbalance is found in contamination levels between intervention and control villages, then this will be adjusted for in final models.

#### Process evaluation

The activity survey and record book data will be descriptively analyzed to assess fidelity and reach of intervention delivery. Preliminary analyses will also be conducted on the activity survey data during implementation to provide Gram Vikas with targeted feedback on areas for improvement. This information will be communicated only to the Gram Vikas management staff who will then relay the feedback to the mobilizer team.

Transcripts from the interviews with Gram Vikas mobilizers and intervention participants will be thematically analyzed. The analysis will focus on themes related to lessons learned on implementation delivery, perceptions of and level of satisfaction with intervention activities, if and how the activities influenced CFM practices, and recommendations for the future. Research team members will first read through each transcript and record memos reflecting on the interview discussion. A codebook will then be developed based on the memos and topics covered in the interview guides, allowing for both inductive and deductive codes. Researchers will then apply codes to the transcripts and subsequently examine coded segments to identify themes related to the topics of interest, but still allowing for unanticipated, yet informative, themes to emerge.

## Reporting harms, auditing, and dissemination plans

We do not anticipate any harm to participants from taking part in this trial and as such, do not have plans for post-trial care or compensation for incurred harm. However, the US-based and India-based research team members are in frequent communication to discuss and address any issues including adverse events during data collection activities. We do not plan to audit the trial.

Research findings will be disseminated via peer-reviewed journals with open access and scientific conferences. Professional writers will not be used for journal submissions. We will also share findings at bi-annual stakeholder meetings facilitated by Gram Vikas and attended by state and district government officials, local academic institutions, and other community development NGOs. At the end of the study we may hold meetings in trial villages to share findings with participants.

## Discussion

We present the protocol for a two-arm CRT with parallel group design to evaluate the effectiveness of a behavior change intervention aimed at improving safe disposal of child feces and child latrine use among rural households in Odisha, India. This study addresses a crucial knowledge gap — there is limited evidence on successful interventions that focus solely on child sanitation practices, are designed using formative research and behavioral theory, and address both safe disposal and latrine training to ensure *all* child feces are safely managed. Results will offer insights for effective CFM behavioral interventions that could be scaled-up in Gram Vikas’s broader programming, integrated into the Government of India’s next iteration of sanitation campaigns, or implemented in other rural contexts with low rates of safe CFM.

A notable factor that has already influenced this study and will continue to is the COVID-19 pandemic. Field activities were paused for a six-month period after baseline data collection as the novel coronavirus spread globally and the Gram Vikas team concentrated their efforts on Odisha’s pandemic response. Being a more isolated area, Gajapati district continues to have a low caseload while Ganjam district, once a viral hotspot, has drastically slowed its infection rate [48, 49]. It is possible the situation could change and research activities will again need to halt, potentially affecting implementation or endline data collection. The COVID-19 pandemic also influenced intervention design where community-wide behavior change strategies were purposefully not considered to safeguard against viral spread. However, as the intervention is meant only for a sub-set of the community (i.e. households with a child < 5 years), such strategies were already not prioritized. Gram Vikas will implement the intervention following preventative measures such as mask-wearing and keeping physical distance from participants. It is this ‘COVID conscious’ version of the intervention that will be evaluated but we do not anticipate such additional measures to impede the behavior change strategies. Finally, there is a potential for the external context of the pandemic to influence caregiver’s CFM practices from a heightened risk perception and promotion of related-behaviors like handwashing. During the pause period we conducted phone interviews among trial villages to assess how COVID-19 impacted WASH practices. While we found the pandemic impacted daily life [50], we found no evidence of impact on CFM or sanitation behaviors [51]; any impact would also equally affect both intervention and control arms.

Beyond the evaluation of the CFM intervention itself, this study will examine a number of innovative research areas. The RANAS approach to systematic behavior change has been used to design interventions on many different WASH behaviors, but this is the first CRT to evaluate its application on safe disposal and child latrine training. We are also testing a latrine training mat with tray, which is a novel hardware that we developed and co-designed with local community members using a user-centered design approach. While this hardware was inspired by the ‘Safe Squat’ mat pilot-tested in Kenya [52], it was modified through extensive formative research for use with pour-flush latrines (the latrine type in our study area). Moreover, the latrine mat includes handles and a tray so it can be safely used both outside and inside of the latrine, enabling use with a broader range of child age groups. The latrine mat will be rigorously evaluated in a trial setting, moving this area of research forward. Lastly, while past observational studies have suggested a link between CFM practices and household fecal contamination [8, 53, 54], this will be the first CRT to evaluate the impact of a dedicated CFM intervention on household levels of fecal contamination.

## Supplementary Information

**Additional file 1.**

**Additional file 2.**

## Data Availability

Not applicable.

## Abbreviations

CFM: child feces management
CRT: cluster randomized trial
RANAS: Risks, Attitudes, Norms, Abilities, Self-regulation
ODK: Open Data Kit
GEE: generalized estimating equations
