# Supplementary material for: Study design and rationale for a cluster randomized trial of a safe child feces management intervention in rural Odisha, India
Source: BMC Public Health. 2022 Jan 15;22:106. doi: 10.1186/s12889-021-12405-0 (PMC8760865; doi:10.1186/s12889-021-12405-0)
Supplement: Supplementary file 1 — Additional file 1. [file 12889_2021_12405_MOESM1_ESM.docx]

**SPIRIT 2013 Checklist:**

**Title:** Study design and rationale for a cluster randomized trial of a safe child feces management intervention in rural Odisha, India

**Authors:**

Gloria D. Sclar^1,2^, Valerie Bauza^1^, Hans-Joachim Mosler^2,3^, Alokananda Bisoyi^4^, Howard H. Chang^1,5^, Thomas F. Clasen^1^

| **Section/ Item** | **Item** | **Description** | **Comments** |
| --- | --- | --- | --- |
| **Administrative information** | | | |
| **Title** | **1** | Descriptive title identifying the study design, population, interventions, and, if applicable, trial acronym | Title page – pg 1 |
| **Trial registration** | **2a** | Trial identifier and registry name. If not yet registered, name of intended registry | Abstract – pg 2;  Background – paragraph 6, pg 4 |
|  | **2b** | All items from the World Health Organization Trial Registration Data Set | Yes – we are registered at ISRCTN which is approved by WHO and includes all items. See row above for where trial registry in ISRCTN is noted in the protocol. |
| **Protocol version** | **3** | Date and version identifier | Not applicable – this is the first version of the protocol |
| **Funding** | **4** | Sources and types of financial, material, and other support | Funding – pg 15 |
| **Roles and responsibilities** | **5a** | Names, affiliations, and roles of protocol contributors | Title page – pg 1;  Author’s contributions – pg 15 |
|  | **5b** | Name and contact information for the trial sponsor | Title page – pg 1 (corresponding author noted) |
|  | **5c** | Role of study sponsor and funders, if any, in study design; collection, management, analysis, and interpretation of data; writing of the report; and the decision to submit the report for publication, including whether they will have ultimate authority over any of these activities | Funding – pg 15 |
|  | **5d** | Composition, roles, and responsibilities of the coordinating centre, steering committee, endpoint adjudication committee, data management team, and other individuals or groups overseeing the trial, if applicable (see Item 21a for data monitoring committee) | Not applicable |
| **Introduction** | | | |
| **Background and rationale** | **6a** | Description of research question and justification for undertaking the trial, including summary of relevant studies (published and unpublished) examining benefits and harms for each intervention | Background - pgs 3 to 4 (all paragraphs help give justification); specific research questions in paragraph 6, pg 4 |
|  | **6b** | Explanation for choice of comparators | Study design – pg 4 |
| **Objectives** | **7** | Specific objectives or hypotheses | Background - paragraph 6, pg 4 |
| **Trial Design** | **8** | Description of trial design including type of trial (eg, parallel group, crossover, factorial, single group), allocation ratio, and framework (eg, superiority, equivalence, noninferiority, exploratory) | Study design – pg 4 |
| **Methods: Participants, interventions, and outcomes** | | | |
| **Study Setting** | **9** | Description of study settings (eg, community clinic, academic hospital) and list of countries where data will be collected. Reference to where list of study sites can be obtained | Study setting and population – pg 5 |
| **Eligibility Criteria** | **10** | Inclusion and exclusion criteria for participants. If applicable, eligibility criteria for study centres and individuals who will perform the interventions (eg, surgeons, psychotherapists) | Inclusion/exclusion criteria – pgs 5 to 6 |
| **Interventions** | **11a** | Interventions for each group with sufficient detail to allow replication, including how and when they will be administered | Interventiong – pgs 6 to 8 |
|  | **11b** | Criteria for discontinuing or modifying allocated interventions for a given trial participant (eg, drug dose change in response to harms, participant request, or improving/worsening disease) | Not applicable (no harm to participation anticipated which is noted in Reporting harms, auditing, and dissemination plans on pg 13) |
|  | **11c** | Strategies to improve adherence to intervention protocols, and any procedures for monitoring adherence (eg, drug tablet return, laboratory tests) | Not applicable (conducting intention-to-treat analysis as noted in Data analysis paragraph 1, pg 12) |
|  | **11d** | Relevant concomitant care and interventions that are permitted or prohibited during the trial | Not applicable |
| **Outcomes** | **12** | Primary, secondary, and other outcomes, including the specific measurement variable (eg, systolic blood pressure), analysis metric (eg, change from baseline, final value, time to event), method of aggregation (eg, median, proportion), and time point for each outcome. Explanation of the clinical relevance of chosen efficacy and harm outcomes is strongly recommended | Study outcomes and measures – pgs 8 to 9 |
| **Participant Timeline** | **13** | Time schedule of enrolment, interventions (including any run-ins and washouts), assessments, and visits for participants. A schematic diagram is highly recommended (see Figure) | Figure 1 |
| **Sample Size** | **14** | Estimated number of participants needed to achieve study objectives and how it was determined, including clinical and statistical assumptions supporting any sample size calculations | Sample size – pg 9 |
| **Recruitment** | **15** | Strategies for achieving adequate participant enrolment to reach target sample size | Data and sampl collection: trial – paragraph 2, pg 10 |
| **Methods: Assignment of interventions (for controlled trials)** | | | |
| **Sequence generation** | **16a** | Method of generating the allocation sequence (eg, computergenerated random numbers), and list of any factors for stratification. To reduce predictability of a random sequence, details of any planned restriction (eg, blocking) should be provided in a separate document that is unavailable to those who enroll participants or assign interventions | Village selection and random allocation – pg 10 |
| **Allocation concealment mechanism** | **16b** | Mechanism of implementing the allocation sequence (eg, central telephone; sequentially numbered, opaque, sealed envelopes), describing any steps to conceal the sequence until interventions are assigned | Village selection and random allocation – pg 10 |
| **Implementation** | **16c** | Who will generate the allocation sequence, who will enrol participants, and who will assign participants to interventions | Village selection and random allocation – pg 10;  Data and sample collection: trial – pgs 10 to 11 |
| **Blinding (masking)** | **17a** | Who will be blinded after assignment to interventions (eg, trial participants, care providers, outcome assessors, data analysts), and how | Intervention: CFM Intervention – Behavior Change Strategies – paragraph 1, pg 7;  Data and sample collection: trial – paragraph 1, pg 10 |
|  | **17b** | If blinded, circumstances under which unblinding is permissible, and procedure for revealing a participant’s allocated intervention during the trial | Not applicable (no blinding) |
| **Methods: Data collection, management, and analysis** | | | |
| **Data collection methods** | **18a** | Plans for assessment and collection of outcome, baseline, and other trial data, including any related processes to promote data quality (eg, duplicate measurements, training of assessors) and a description of study instruments (eg, questionnaires, laboratory tests) along with their reliability and validity, if known. Reference to where data collection forms can be found, if not in the protocol | Data and sample collection – pgs 10 to 11 |
|  | **18b** | Plans to promote participant retention and complete follow-up, including list of any outcome data to be collected for participants who discontinue or deviate from intervention protocols | Not applicable |
| **Data Management** | **19** | Plans for data entry, coding, security, and storage, including any related processes to promote data quality (eg, double data entry; range checks for data values). Reference to where details of data management procedures can be found, if not in the protocol | Data management – pgs 11 to 12 |
| **Statistical methods** | **20a** | Statistical methods for analysing primary and secondary outcomes. Reference to where other details of the statistical analysis plan can be found, if not in the protocol | Data analysis – pgs 12 to 13 |
|  | **20b** | Methods for any additional analyses (eg, subgroup and adjusted analyses) | Data analysis – paragraphs 1 (trial) and 2 (environmental sampling), pgs 12 to 13 |
|  | **20c** | Definition of analysis population relating to protocol non-adherence (eg, as randomised analysis), and any statistical methods to handle missing data (eg, multiple imputation) | Data analysis – paragraph 1 (trial), pg 12 |
| **Data monitoring** | **21a** | Composition of data monitoring committee (DMC); summary of its role and reporting structure; statement of whether it is independent from the sponsor and competing interests; and reference to where further details about its charter can be found, if not in the protocol. Alternatively, an explanation of why a DMC is not needed | Not applicable |
|  | **21b** | Description of any interim analyses and stopping guidelines, including who will have access to these interim results and make the final decision to terminate the trial | Not applicable |
| **Harms** | **22** | Plans for collecting, assessing, reporting, and managing solicited and spontaneously reported adverse events and other unintended effects of trial interventions or trial conduct | Reporting harms, auditing, and dissemination plans – paragraph 1, pg 13 |
| **Auditing** | **23** | Frequency and procedures for auditing trial conduct, if any, and whether the process will be independent from investigators and the sponsor | Reporting harms, auditing, and dissemination plans – paragraph 1, pg 13 |
| **Ethics and dissemination** | | | |
| **Research ethics approval** | **24** | Plans for seeking research ethics committee/institutional review board (REC/IRB) approval | Ethics approval and consent to participate – pg 15 |
| **Protocol amendments** | **25** | Plans for communicating important protocol modifications (eg, changes to eligibility criteria, outcomes, analyses) to relevant parties (eg, investigators, REC/IRBs, trial participants, trial registries, journals, regulators) | Ethics approval and consent to participate – pg 15 |
| **Consent or assent** | **26a** | Who will obtain informed consent or assent from potential trial participants or authorised surrogates, and how (see Item 32) | Data and sample collection – pgs 10 to 11;  Ethics approval and consent to participate – pg 15 |
|  | **26b** | Additional consent provisions for collection and use of participant data and biological specimens in ancillary studies, if applicable | Not applicable |
| **Confidentiality** | **27** | How personal information about potential and enrolled participants will be collected, shared, and maintained in order to protect confidentiality before, during, and after the trial | Data management – pgs 11 to 12 |
| **Declaration of interests** | **28** | Financial and other competing interests for principal investigators for the overall trial and each study site | Competing interests – pg 15 |
| **Access to data** | **29** | Statement of who will have access to the final trial dataset, and disclosure of contractual agreements that limit such access for investigators | Data management – final sentence, pg 12  There is no contractual agreements that limit access for investigators |
| **Ancillary and post-trial care** | **30** | Provisions, if any, for ancillary and post-trial care, and for compensation to those who suffer harm from trial participation | Reporting harms, auditng, and dissemination plans – paragraph 1, pg 13 |
| **Dissemination policy** | **31a** | Plans for investigators and sponsor to communicate trial results to participants, healthcare professionals, the public, and other relevant groups (eg, via publication, reporting in results databases, or other data sharing arrangements), including any publication restrictions | Reporting harms, auditng, and dissemination plans – paragraph 2, pg 13 |
|  | **31b** | Authorship eligibility guidelines and any intended use of professional writers | Reporting harms, auditng, and dissemination plans – paragraph 2, pg 13 |
|  | **31c** | Plans, if any, for granting public access to the full protocol, participant level dataset, and statistical code | Availability of data and materials – pg 15 |
| **Appendices** | | | |
| **Informed consent materials** | **32** | Model consent form and other related documentation given to participants and authorized surrogates | Ethics approval and consent to participate – pg 15 (available upon request) |
| **Biological** **Specimens** | **33** | Plans for collection, laboratory evaluation, and storage of biological specimens for genetic or molecular analysis in the current trial and for future use in ancillary studies, if applicable | Not applicable |
